# Protective Effect of Genistein on Condylar Cartilage through Downregulating NF-*κ*B Expression in Experimentally Created Osteoarthritis Rats

**DOI:** 10.1155/2019/2629791

**Published:** 2019-12-30

**Authors:** Jian Yuan, Wanghui Ding, Na Wu, Shijie Jiang, Wen Li

**Affiliations:** ^1^The Affiliated Stomatology Hospital, Zhejiang University School of Medicine, Hangzhou 310006, China; ^2^Key Laboratory of Oral Biomedical Research of Zhejiang Province, Zhejiang University School of Stomatology, Hangzhou 310006, China

## Abstract

Temporomandibular joint osteoarthrosis (TMJOA) is characterised by chronic inflammatory changes, with subsequent gradual loss of joint cartilage. NF-*κ*B is a crucial transcription factor in the course of inflammatory and immune responses, which are involved in OA pathology activated by proinflammatory cytokines. Genistein is known to have anti-inflammation and modulation of metabolic pathways through repression of the NF-*κ*B signaling pathway in inflammatory disease. But so far, studies on the effects of genistein on TMJOA are very limited. So, the purpose of this study is to investigate the protective effect of genistein against experimentally induced condylar cartilage degradation through downregulating NF-*κ*B expression in created osteoarthritis rats *in vivo*. Male SD rats were created as temporomandibular joint osteoarthritis models and administered through oral gavage with low and high dosage genistein (30 mg/kg and 180 mg/kg, respectively) daily for 4 weeks. The morphological changes of the condylar cartilage were studied with HE and Masson staining. The expressions of p65 and inflammatory cytokines (IL-1*β* and TNF*α*) were detected using immunohistochemistry and real-time PCR. The results showed that experimentally created osteoarthritis reduced the condylar cartilage thickness of rats and increased the gene expression of cytokines (IL-1*β* and TNF*α*) and positive cells of p65. Genistein treatment had positive effects on the condylar cartilage renovation, while high dose genistein treatment had more significant effects on the reversing of OA changes and reduction of the expression of p65 and inflammatory cytokines (IL-1*β* and TNF*α*). The results indicated that high dose genistein treatment had obvious therapeutic effects on condyle cartilage damages of OA rats. The mechanism may be that genistein suppresses the NF-*κ*B expression activated by inflammatory cytokines.

## 1. Introduction

Temporomandibular joint osteoarthrosis (TMJOA), which is characterised by chronic inflammatory changes, with subsequent gradual loss of joint cartilage, mostly occurred in condyle [1–3]. Although condyle degradation can cause many clinical symptoms such as arthralgia, joint clicking, mandibular deviation, or retraction, it can hardly be radically cured because the exact etiologies of TMJOA were not fully understood [4, 5]. However, inflammatory pathways were verified as a “triggering factor” that activated the immune responses in OA pathogenesis [6]. Proinflammatory cytokines including interleukin-1*β* (IL-1*β*) and tumor necrosis factor-*α* (TNF-*α*) played a significant role in the development of OA. Some studies showed that IL-1*β* and TNF-*α* contributed to the pathogenesis of osteoarthrosis and induced the inflammation and destruction of the joints [7,8]. IL-1*β* was reported to involve in the activation of the inflammatory pathways in synovitis and cartilage degradation. Meanwhile, TNF-*α* is also a potent proinflammatory cytokine which had higher expression in the OA joints and participated in host immune response [7, 8].

Nuclear factor *κ*B (NF-*κ*B) has been considered as a central mediator in diverse cellular responses, particularly in the inflammatory process and immune response [9]. Many studies reported that IL-1*β* and TNF-*α* induced the nuclear translocation of NF-*κ*B [10–14]. Then, NF-*κ*B activated the downstream Bcl-2 family proteins to regulate apoptosis [15, 16]. Bcl-2 and Bax are both important members in the Bcl-2 family, which involved in the mechanism of apoptotic cell death [17].

Genistein is an isoflavone compound extracted from plants, well known for its anti-inflammatory, antioxidant, and anticancer effects, is widely used for the treatment of inflammatory diseases [18]. Some study has demonstrated that genistein has a bone-protective effect in rat models of osteoporosis in knee joints [19]. However, the knee and hip joints are composed of hyaline cartilage, while mandibular condyle is covered by fibrocartilage [20]. Up to now, the studies about its therapeutic effect on TMJOA is limited.

In this study, we investigated the therapeutic effect and the possible mechanism of genistein on OA of TMJ in rats and presumed that genistein has restorative effects on cartilage destruction by inhibition of NF-kB signaling pathways.

## 2. Materials and Methods

### 2.1. Model Building

**Figure 1:**
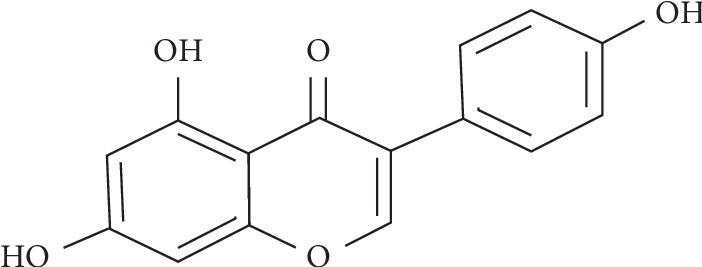
Chemical structure of genistein.

Twenty-four male rats (8-week old, 232.3 ± 2.5 g) were purchased from the animal center of Zhejiang University. The rats were divided into a normal control group (NC), four-week OA group (OA), low-dose GE group (GE1), and high-dose GE group (GE2). The setup of the TMJOA model was referred to previous studies [21,22]. Collagenase (0.05 ml; Sigma Biochemical, St. Louis) was injected in the upper cavity of TMJ in OA, GE1, and GE2 groups, and normal saline (0.05 ml) was injected in the NC group. All animals were given substitute diet with corn oil replacing soybean oil to avoid the interference of GE in soybean [22]. Daily treatment was performed with genistein (99.5% pure; Winherb Med., China) and sterile saline by intragastric administration in GE1 (30 mg/kg), GE2 (180 mg/kg), NC (2 ml saline), and OA (2 ml saline) groups, respectively.  Figure 1 showed the chemical structure of genistein [23].

### 2.2. Measurement of Cytokines by RT-PCR

Synovial fluid samples were collected from the superior joint cavity using diluted aspiration as described by Uehara et al. [24]. Briefly, 2% thiopental sodium anaesthesia (35 mg/kg) was injected after disinfection. 0.2 ml saline was repeatedly injected and withdrawn in the upper joint cavity to aspirate synovial fluids. The mean diluted synovial fluid was 0.20 ml (range: 0.16–0.24 ml). The aspirates were centrifuged at 3000 ×*g* for 15 min at room temperature, and the supernatant was kept for RT-PCR. Total RNA was extracted using an RNA mini kit (BIO-RAD, USA) according to the manufacturer. The cDNA of various groups were synthesized according to the GeneAmp PCR kit (ABI, USA). RT-PCR was performed by using a SYBR green RT-qPCR kit (TOYOBO Corporation). All PCR reactions were performed using iCycler iQTM (Bio-Rad, Hercules, CA, USA). The cycling conditions were 10 min at 95°C, followed by 40 cycles: denaturation at 94°C for 15 s, annealing for 30 s at 57°C, and extension at 72°C for 30 s. The primers for IL-1*β* were F: 5′-CAC CTT CTT TTC CTT CAT CTT TG-3′ and R: 5′-GTC GTT GCT TGT CTC TCC TTG TA-3′, the product length is 241, and the GenBank Accession Number is NM_031512.2; for TNF-*α* were F: 5′-ACT GAA CTT CGG GGT GAT TG-3′ and R: 5′-GCT TGG TGG TTT GCT ACG AC-3′, the product length is 153, and the GenBank Accession Number is XM_008772775.2; and for GAPDH were F: 5′-GTA TTG GGC GCC TGG TCA CC-3′ and R: 5′-CGC TCC TGG AAG ATG GTG ATG G-3′, the product length is 202, and the GenBank Accession Number is XM_017593963.1.

### 2.3. Histopathology and Immunohistochemistry

The haematoxylin and eosin (HE) and Masson staining were operated according to previous studies [21]. After synovial fluid collection, all the rats were sacrificed. Condyles were dissected carefully, fixed in 4% PFA, then decalcified in 10% EDTA for 4 weeks. After dehydration, the samples were incubated in paraffin overnight. The left condyles were prepared for sagittal sections and stained with HE and Masson, respectively. The destruction of the joint cartilage was evaluated by the modified Mankin score system. Three independent location of samples were chosen for assessment [25]. The description for histological evaluation included 4 aspects: structural integrity (0–6 points)(HE), matrix staining (0–4 points)(Masson), cellularity (0–3 points)(HE), and tidemark integrity (0–2 points)(HE) [25]. The right condyles were prepared for immunohistochemistry of p65. After dewaxing by immersing in xylene, the samples were incubated with primary monoclonal mouse anti-p65 antibody (Abcam, USA; dilution 1 : 40) for 1 h at 37°C. The percentage of 65 positive chondrocytes was calculated by the number of immune positive cell divided by total cell number. All sections (*n* = 3) were analyzed under the microscope (Olympus IX71, Japan).

### 2.4. Statistical Analysis

The data are expressed as mean ± SD. SPSS 16.0 was used for the statistical analysis. Also, *p* values less than 0.05 were considered to have a statistically significant difference. One-way ANOVA was used for multiple comparisons among various groups.

## 3. Results and Discussion

### 3.1. Results

#### 3.1.1. Structural Changes in the Condyle Cartilage

**Figure 2:**
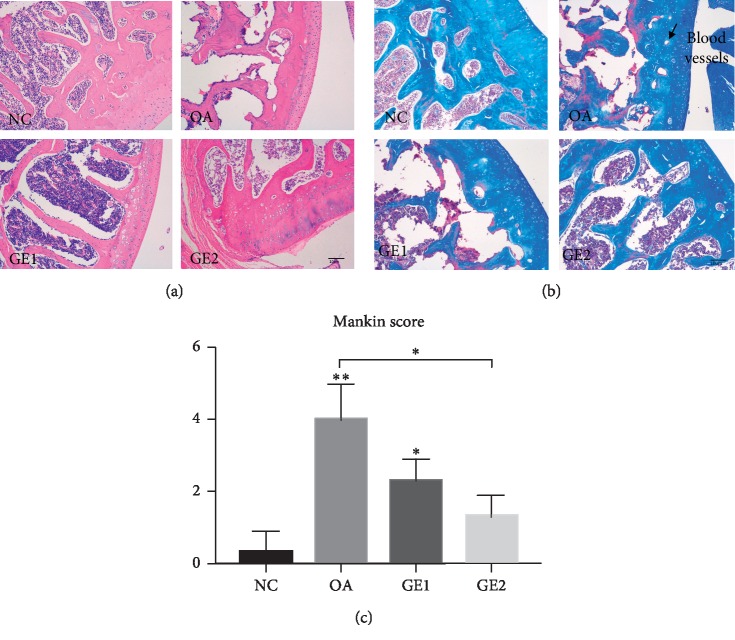
Treatment effects of GE on condyle cartilage changes in the rat model of TMJOA. (a) Representative histology changes in the condyle cartilage for the OA and GE treatment animals at 4 weeks after injection by HE staining. (b) Demonstration of matrix changes by Masson staining. (c) Quantitation of histology changes by the modified Mankin score system. The histological evaluation included 4 aspects. HE staining for the evaluation of structural integrity, cellularity, and tidemark integrity and Masson-stained sections for the evaluation of the cartilage matrix. Quantitative data are shown as means ± SD. ^*∗*^*p* < 0.05, ^*∗∗*^*p* < 0.01, significantly different when compared with NC and OA groups, respectively, *n* = 6.

HE staining of the condyle cartilage in various groups is shown in Figure 2. Compared with the NC group, significant changes of the cartilage were found in the OA group. The cartilage thickness of OA rats reduced obviously compared with the NC group. Also, multilevel cartilage tidemarks appeared in the OA group. There were also some changes in the bone trabecula and marrow cavity. OA animals had narrowed bone trabecula and bigger marrow cavity, while GE treatment had restorative effects on condylar cartilage repairment. High-dose genistein treatment showed more obvious improvement on cartilage repairment than low-dose treatment. Masson staining showed the same tendency of structural changes with HE staining. In OA animals, several blood vessels and cell mass appeared, while GE treatment could reduce this kind of changes. The modified Mankin scores system was used to evaluate the structural changes of the condyle cartilage in the four groups. A statistically significant difference in the Mankin score was observed in the NC, OA, and GE treatment groups. The score of OA was higher than that of NC and GE treatment groups. High-dose genistein treatment showed more improvement on cartilage repairment.

#### 3.1.2. Immunohistochemistry Analysis of p65 in Condyle Cartilage

**Figure 3:**
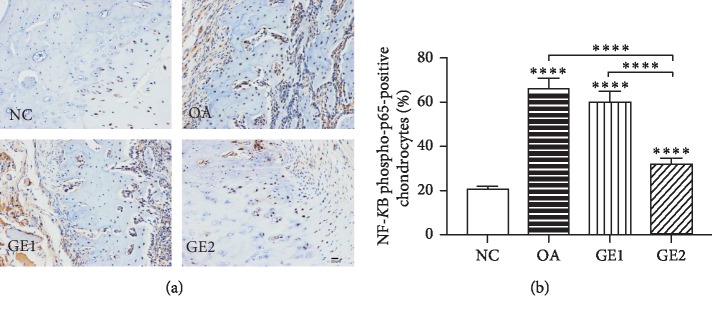
Effects of GE on P65 immunoreactivity in TMJOA rats. (a) GE inhibits p65 immunoreactivity in the condylar cartilage of OA rats. (b) Comparative intensity of p65 in various groups. Quantitative data are shown as means ± SD. ^*∗*^*p* < 0.05, ^*∗∗*^*p* < 0.01, ^*∗∗∗*^*p* < 0.001, ^*∗∗∗∗*^*p* < 0.0001, significantly different when compared with NC and OA groups, respectively, *n* = 6.

p65 immunoreactivity can be found in all positive sections of the cartilage cells throughout the condylar cartilage (Figure 3(a)). Positive cells were identified as tissue with brown staining. Comparative intensity of p65 in various groups is shown in Figure 3(b), which displayed marked variation in various groups. Positive signals of p65 in the OA group was much more than that in the NC group (*p* < 0.0001), while high-dose GE treatment significantly reduced the intensity of p65 (*p* < 0.0001).

#### 3.1.3. Expression of IL-1*β* and TNF-*α* in the Condylar Cartilage

**Figure 4:**
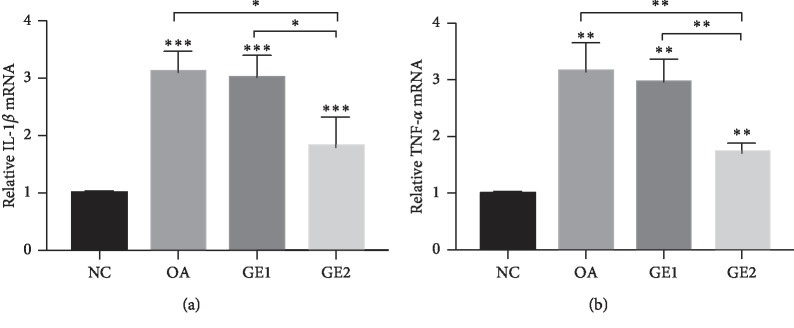
Effects of GE on the gene expression of IL-1*β* and TNF-*α*. (a) IL-1*β* expression in OA animals increased significantly compared with the NC group, while high dose of GE could significantly inhibit IL-1*β* expression. (b) TNF-*α* expression increased significantly in OA animals, and high dose of GE treatment could significantly inhibit TNF-*α* expression. Quantitative data are shown as means ± SD. ^*∗*^*p* < 0.05, ^*∗∗*^*p* < 0.01, ^*∗∗∗*^*p* < 0.001, significantly different when compared with NC and OA groups, respectively, *n* = 6.

The results of the real-time PCR analysis are shown in Figure 4. In the OA group, the expression of IL-1*β* was much higher than that in the NC group (*p* < 0.001), and the expression of TNF-*α* had the same tendency with IL-1*β* (*p* < 0.01). Meanwhile, low-dose GE treatment could decrease the expression of IL-1*β* and TNF-*α*, but significantly statistical difference was not achieved (*p* > 0.05), while the high-dose GE treatment significantly reduced the expression of inflammatory factors (*p* < 0.01).

## 4. Discussion

This is the first study to investigate the effect of genistein on cartilage repairment of temporomandibular joint osteoarthritis models *in vivo*. In this study, the TMJOA model was set up on rats by injection of collagenase [21, 22], which causes histological changes including erosion of the cartilage surface, decreased thickness of the cartilage, and even changes in the bone trabecula and marrow cavity. In the GE treatment groups, the high-dose GE had better therapeutic effects compared with low GE groups, which are as indicated by Mankin scores. Masson staining of the present study showed the same tendency of structural changes with HE staining in various groups, which verified the therapeutic effects of GE.

The destruction of the extracellular matrix is usually accompanied by an increase of proinflammatory cytokines, such as IL-1*β* and TNF-*α* and these cytokines are also key mediators of the intracapsular pathological conditions of the temporomandibular joint [7]. IL-1*β* plays a critical role in OA pathogenesis, which was verified independently or combining with other mediators to induce inflammatory reactions and catabolic effect in the course of OA [12, 13]. Many studies demonstrated that patients with OA have an elevated level of IL-1*β* than normal people [13], and blocking IL-1*β* production could counteract the degradative mechanisms associated with OA pathology [26]. Our result agreed with the previous publications. The expression of IL-1*β* was higher in OA rats than that of normal rats. Meanwhile, TNF*α* is another key inflammatory cytokine in the pathophysiological processes of OA. Many studies reported that synovial fluids of OA joints showed higher concentration of TNF*α* than that of normal joints [7, 24, 27], while high TNF*α* expression was detected in local arthritic tissues, which was related to the autoimmune reaction and can cause aggressive cartilage destruction of joints by suppressing the synthesis of the cartilage matrix [24]. The present result showed that the expression of TNF-*α* was higher in OA rats than that of normal rats, which had the same tendency with IL-1*β*.

NF-*κ*B is a crucial transcription factor that participates in the development of inflammatory and immune diseases activated by inflammatory responses [12, 28]. In an inactive state, NF-*κ*B binds to the I-*κ*B member. Some inflammatory cytokines could activate I-*κ*B*α*, and then p65 protein separates from I-*κ*B*α* and translocates from cytoplasm to the nucleus to bind related inflammation genes to induce inflammation [18]. Many studies provide evidence for the role of NF-*κ*B in mediating enhancement of apoptosis [14, 15]. Apoptosis, or programmed cell death, has been suggested to have a close relationship with OA progression [29,30]. In the adult of TMJ dysfunction, apoptosis has been found in the course of joint remodeling [31]. Emerging evidence suggests apoptosis and NF-*κ*B signaling are highly activated in OA pathology which can be triggered by proinflammatory cytokines [13, 14, 29]. These inflammatory mediators were verified to activate NF-*κ*B signaling pathways abnormally to induce apoptosis in OA chondrocytes [12]. The present study showed that positive signals of p65 in various groups have the same tendency with the expressions of inflammatory mediators, which indicated that NF-*κ*B phosphorylation may be induced by IL-1*β* and TNF-*α* in the TMJOA model.

Genistein, which is known to have multiple molecular effects, such as the inhibition of inflammation, promotion of apoptosis, and modulation of metabolic pathways, plays an important role in preventing and treating common disorders [23]. Studies on positive effects of genistein have been reported in inflammatory disease. Genistein had positive effect on the treatment of psoriasis by inhibiting TNF-*α*-induced I-kB*α* phosphorylation and p65 nuclear translocation [18]. Genistein was also shown to suppress the production of COX-2 and NO in primary human chondrocytes [32]. Some study indicates that genistein could reverse ox-LDL-induced inflammation through repression of the NF-*κ*B signaling pathway in HUVECs [33]. But so far, studies on the effects of genistein on TMJOA are very limited. The present study found that high-dose GE treatment had obviously therapeutic effects on condyle cartilage damage of OA rats. Furthermore, high-dose GE treatment could reduce positive signals of p65 and the expression of inflammatory mediators in TMJOA rats, which indicated that the effects of genistein on repairing cartilage damage may be through the repression of IL-1*β*-TNF-*α*-mediated NF-*κ*B signaling pathway in TMJOA models. It is recognized that Bcl-2 and Bax play a crucial role in the regulation of the apoptotic process [20, 31]. The effect of genistein on cartilage repairment may be through repression of the NF-*κ*B signaling pathway, which in turn decreased the ratio of Bax/Bcl-2. Another mechanism may be related to hypoxia-inducible factor-2 alpha (HIF-2*α*) which was regulated by NF-*κ*B before and during inflammation [34]. HIF-2*α* downstream degradation factors, such as MMP-3,13 and ADAMTs-4,5, played essential roles in the degradation of cartilage aggrecan and had been recognized as one of the most primary targets for therapeutic intervention in osteoarthritis [21]. However, further investigation on the underlying mechanism of the effect of genistein on the cartilage repairment of TMJOA models is still needed.

## 5. Conclusions

This is the first study to investigate the protective effect of genistein on cartilage repairment of temporomandibular joint osteoarthritis models *in vivo*. This study revealed that high-dose GE treatment had obviously therapeutic effects on cartilage repairment and downregulated the expression of p65 and the inflammatory mediators. It was indicated that therapeutic effects of genistein may be through repression of the NF-*κ*B signaling pathway.

## Data Availability

The data that support the findings of this study are available from the corresponding author upon request.
